# Extensive Variations in Diurnal Growth Patterns and Metabolism Among *Ulva* spp. Strains[Author-notes fn1][Author-notes fn6]

**DOI:** 10.1104/pp.18.01513

**Published:** 2019-02-12

**Authors:** Antoine Fort, Morgane Lebrault, Margot Allaire, Alberto A. Esteves-Ferreira, Marcus McHale, Francesca Lopez, Jose M. Fariñas-Franco, Saleh Alseekh, Alisdair R. Fernie, Ronan Sulpice

**Affiliations:** a National University of Ireland - Galway, Plant Systems Biology Lab, Ryan Institute, Plant and AgriBiosciences Research Centre, School of Natural Sciences, Galway H91 TK33, Ireland; b National University of Ireland - Galway, Genetics and Biotechnology Lab, Ryan Institute, Plant and AgriBiosciences Research Centre, School of Natural Sciences, Galway H91 TK33, Ireland; c Max Planck Institute of Molecular Plant Physiology Am Mühlenberg, 14476 Potsdam-Golm, Germany; d Center of Plant System Biology and Biotechnology, 4000 Plovdiv, Bulgaria

## Abstract

Green macroalgae of the genus *Ulva* play a key role in coastal ecosystems and are of increasing commercial importance. However, physiological differences between strains and species have yet to be described in detail. Furthermore, the strains of *Ulva* used in aquaculture usually originate from opportunistic collection in the wild without prior selection of best performing strains. Hence, efforts are required to detect the potential variability in growth and metabolic accumulation between *Ulva* strains and ultimately select the best performing strains under given environmental conditions. Here, the growth, physiological, and metabolic characteristics of 49 laminar *Ulva* spp. strains were investigated using a custom-made high-throughput phenotyping platform, enzymatic assays, and gas chromatography-mass spectrometry. We found large natural variation for a wide range of growth and metabolic characteristics, with growth rates varying from 0.09 to 0.37 mg.mg^−1^.d^−1^ among strains. *Ulva* spp. possess a unique diurnal growth pattern and primary metabolism compared with land plants, with higher growth rates during the night than during the light period. Starch and sucrose only contributed on average 35% of the carbon required to sustain *Ulva*’s night growth. Nitrates accumulated during the night in *Ulva* tissues, and nitrate accumulation and consumption was positively correlated with growth. In addition, we identified six amino acids as possible biomarkers for high growth in *Ulva*. The large variability in growth and metabolite accumulation recorded among morphologically similar *Ulva* strains justifies future efforts in strain selection for increasing biomass, metabolite yields, and nutrient removal in the growing aquaculture industry.

## Teaser

Sea lettuce (Ulva spp.) strains show extensive variation in growth and metabolism, demonstrating a large potential for strain selection in boosting biomass and metabolite yields in aquaculture.

The genus *Ulva* represents a large group of green macroalgae with 131 taxonomically accepted species (Guiry et al., 2014). *Ulva* spp. play a key role in coastal ecosystems, contributing to nutrient cycling, and providing food and habitats for a variety of species (Wilson et al., 1990; Human et al., 2015). Although *Ulva* spp. provide important ecosystem services, their rapid growth under certain conditions can lead to green tide events of deleterious ecological and economic consequences (Valiela et al., 1997; Keesing et al., 2011). In addition to their ecological importance, onshore and offshore cultivation of *Ulva* spp. represents an emergent industry of economic importance due to its broad range of potential applications (Bolton et al., 2016), from human consumption and animal feed production (Ortiz et al., 2006; Tabarsa et al., 2012) to bioremediation (Nielsen et al., 2012), and integrated multitrophic aquaculture (Marinho et al., 2013; Silva et al., 2015).

Vegetative blade growth (i.e. nonmeristematic) occurs in both *Ulva* spp morphotypes (i.e. filamentous and laminar; Tan et al., 1999; Miladi et al., 2018) allowing for growth experiments under controlled conditions using excised blade tissues (Wichard et al., 2015). However, although growth responses in laminar *Ulva* spp. to varying environmental conditions have been well documented in laboratory studies (Björnsäter and Wheeler, 1990; Gordillo et al., 2001; Ale et al., 2011; Angell et al., 2014), less attention has been given to the contribution of genetic factors at the exception of, for example, Lawton et al. (2013), which compared eight laminar *Ulva* strains belonging to two species.

Cultivation of *Ulva* spp. relies on opportunistic collection of strains close to the farming site (Silva et al., 2015; Korzen et al., 2016). Therefore, strain selection for genetic variants of *Ulva* species could potentially lead to significant yield increases if there were a large natural variation in yield, and thus boost their potential as viable crops. In addition to biomass increases, intra- and interspecific genetic variability could potentially lead to drastic changes in other traits of agronomic interest, as highlighted by genome wide association studies in plants, such as for starch and protein content (Pasam et al., 2012), metabolite accumulation (Riedelsheimer et al., 2012), or biomass production (McKown et al., 2014).

Most plants and algae rely solely on light energy to reduce carbon dioxide and produce complex organic compounds, such as carbohydrates and amino acids. Therefore, autotrophic organisms are limited in their carbon fixation by the length of the day period. During the night, those photoassimilates are degraded to supply the cells with the carbon and energy required for metabolism and growth (Sulpice et al., 2014). Although this basic mechanism is conserved among the breadth of autotrophic organisms, the types of photoassimilates used for short and/or long-term storage varies extensively between species. Among these strategies, cyanobacteria accumulate carbon for their night requirements in the form of glycogen (Schwarz et al., 2013) and brown algae (Phaeophyceae) are likely to use mannitol and laminarin (Gravot et al., 2010; Michel et al., 2010), whereas land plants can store their carbon reserves as sucrose (Sturm and Tang, 1999), starch (Sulpice et al., 2009), and/or fructans (Vijn and Smeekens, 1999), depending on the species considered. *Ulva* was shown to accumulate starch (Korzen et al., 2016), but whether starch represents the main carbon storage compound in this species remains to be investigated. An efficient carbon storage and remobilization mechanism is essential to sustain the growth of autotrophic organisms during the night period (Graf et al., 2010), and to avoid carbon starvation (Graf and Smith, 2011; Sulpice et al., 2014), especially in the face of changing photoperiods during the growing season.

In this paper, we aimed to investigate the putative inter- and intraspecific growth and metabolic variation among laminar *Ulva* spp., and to highlight interactions between morphological characteristics, metabolites, and growth. Indeed, the domestication of *Ulva* for aquaculture is still in its infancy and more knowledge is needed to better understand (and exploit) the extent of variation in growth/metabolite accumulation between *Ulva* individuals. Hence, we determined the growth pattern of 49 strains of laminar *Ulva* spp., representing six distinct species, collected across Ireland and the Netherlands, along with their morphological and metabolic characteristics. Metabolic traits and growth rates were determined during the day and night to provide information about the diurnal regulation of metabolism and growth in *Ulva spp*. We highlight major differences in metabolic and growth patterns in *Ulva* spp. compared with land plants, and demonstrate that *Ulva* does not rely on starch nor sucrose for its night carbon reserves. In addition, our results, which show extensive variation in growth rate and metabolic composition between the strains investigated, confirm the economic potential of strain selection efforts in streamlining the aquaculture production cycle. Finally, we describe a novel platform that allows for monitoring the growth of hundreds of *Ulva* tissue samples to facilitate the investigation of a wide range of genetic and/or environmental factors.

## RESULTS

### Validation of the Phenotyping Platform

**Figure 1.**
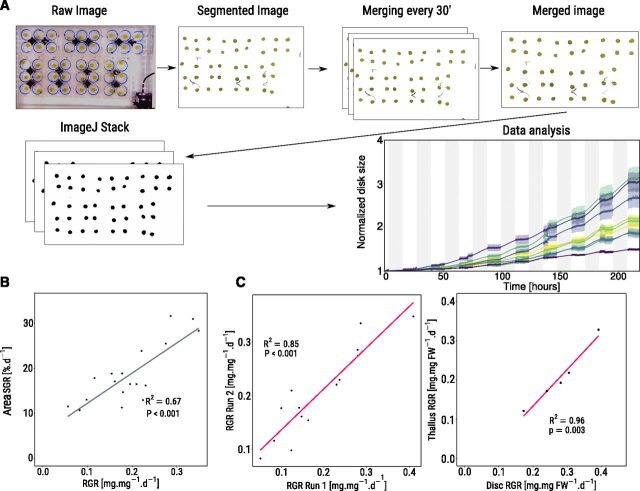
The high throughput growth monitoring platform gives reliable data. A, Image analysis. Details available in “Materials and Methods”. B, Pearson correlation between Area SGR daily and RGR for a subset of 19 *Ulva* strains. C, Reproducibility of the results. Left: RGR daily correlations between the same set of strains, grown after 1 month. Right: correlation between thallus growth and disc growth for five strains.

The quality of the phenotyping platform (Fig. 1A) was assessed by growing 19 strains of *Ulva* spp. in the conditions described in the “Materials and Methods” section, using 8 discs per strain, randomly positioned across the tanks. We then compared the results obtained from the two-dimensional (2D) imaging with biomass accumulation per day. There was a significant, positive correlation between tissue expansion (area specific growth rate, [area SGR]) and the biomass accumulation (relative growth rate [RGR]; R^2^ = 0.67, Pearson correlation, *P* < 0.001; Fig. 1B), indicating that our 2D imaging system can be used to accurately monitor the growth of *Ulva*. To test the reliability of the platform, we repeated the experiment on the exact same strains a month later (excluding four strains that sporulated). We found strong, significant correlations between the RGR of repeated experiments (R^2^ = 0.85; Pearson correlation, *P* < 0.001; Fig. 1C). This indicates that the growth monitoring platform can be used to reliably measure the growth of *Ulva* spp. in replicated experiments and provide repeatable results even after long-time intervals.

In addition, we tested whether 1-week acclimation under the moderate light regime was sufficient to acclimate the strains by comparing the growth of discs from a single strain after 0, 1, and 2 weeks of acclimation under the light-emitting diode (LED) lights. We found no difference in RGR between the three acclimatory regimes (one-way ANOVA, *P* = 0.8; Supplemental Fig. S1), indicating that variations in acclimation periods under moderate light are unlikely to affect growth. Next, we questioned whether disc growth rate was a good proxy for *Ulva* growth by comparing the growth of thalli and discs of five *Ulva* strains. We found that RGR of discs were ∼50% higher than those of thalli for the same strains, indicating a possible growth stimulation effect following wounding. However, we also found a strong positive correlation between thallus and disc growth (R^2^ = 0.96; Fig. 1C), demonstrating that *Ulva* discs can be used as a fast screening method for thallus growth.

Taken together, these results indicate that the methods described here for growth monitoring of dozens of *Ulva* strains in a short time frame (7 d) produce robust and reliable data to compare growth and metabolic characteristics among *Ulva* strains.

### Genetic Characterization of the *Ulva* spp. Strains

**Figure 2.**
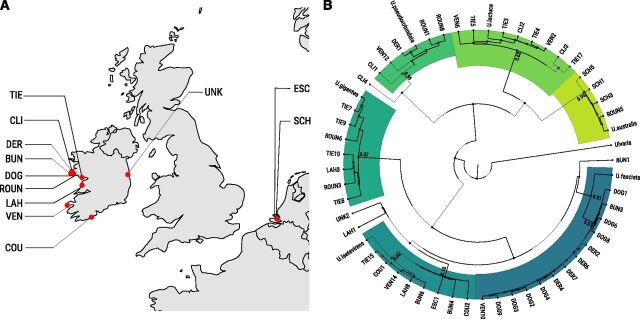
Locations and genetic diversity among the 49 strains tested in this study. Sampling sites (A; see Supplemental Table S1) and phylogenetic tree (B) were obtained using *RbcL* and *tufA* sequences under a GMYC model. Support values for species clusters are indicated on the tree.

Barcoding was used to characterize the 49 individual strains of *Ulva* spp. from Ireland and the Netherlands (Fig. 2A; Supplemental Table S1). We used two barcodes, *RbcL* and *tufA* (Heesch et al., 2009; Saunders and Kucera, 2010), to match each of the 49 strains to its corresponding species, in conjunction with a General Mixed Yule-Coalescent (GMYC) model for species delimitation. The phylogenetic tree from the sequencing results of both RbcL and tufA, using *Ulvaria* spp. sequences as an outgroup, is displayed in Figure 2B. The strains investigated in this study fell into six distinct species: *Ulva australis*, *Ulva gigantea*, *Ulva lactuca*, *Ulva pseudorotundata*, *Ulva laetevirens*, and *Ulva fasciata*. Four strains could not be attributed to a specific species (CLI4, UNK2, LAH1, and BUN1).

### Extensive Inter- and Intraspecific Growth Differences between *Ulva* spp. Strains

**Figure 3.**
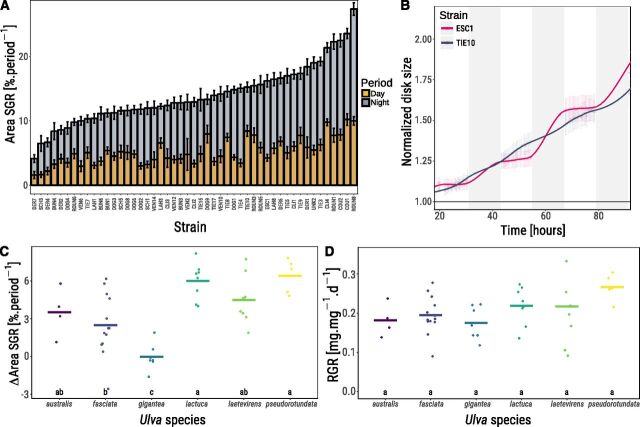
Extensive growth differences between the 49 *Ulva* strains. A, Stacked bar chart representing the mean Area SGR ± sem of each strain; *n* = 12 to 18 discs per strain. B, Example of diurnal pattern growth differences between two strains. Normalized disc size represents the ratio between the disc at the given time point and the starting disc size. Data represent the mean ± sem; *n* = 12 to 18 discs. Night is represented by shaded bars. C, Area SGR difference between night and day among the 49 strains, with their species identification based on the GMYC model. Each dot represents the mean of a given strain, with the median per species shown as a horizontal bar. Letters indicate significance groups among species (Nested ANOVA, *P* < 0.05). D, Same as C but with RGR data.

To investigate the possible differences controlling growth in laminar *Ulva* spp*.,* the tissue expansion (Area SGR) and biomass accumulation (RGR) of 49 individual strains from Ireland and the Netherlands was investigated (Fig. 2A; Supplemental Dataset 1). Daily Area SGR varied from 2.5% to 24% among the 49 strains investigated, with a coefficient of variation (CV) of 37.5%. During the 12-h daytime period, Area SGR values varied between strains from 1.6% to 10% of tissue expansion (CV of 41.11%), whereas we observed higher tissue expansion rates during the night period for most strains, with an Area SGR ranging from 2.5% for the slowest growing strains up to 17% for the fastest growing ones and a CV of 37.8% (Fig. 3A). The tissue expansion pattern was, however, strongly strain dependent, with strains such as TIE10 showing similar daytime and nighttime tissue expansion rates (Fig. 3B). In addition, *U*. *gigantea* strains showed the least day-night variations in Area SGR, whereas *U*. *pseudorotundata* and *U*. *lactuca* strains showed the highest (6% more Area SGR at night than during daylight), suggesting that the day/night tissue expansion patterns observed in *Ulva* might be species specific (Fig. 3C). In addition, comparing Area SGRs by species yielded significant differences, with *U*. *australis* and *U*. *fasciata* displaying a lower tissue expansion during the 24-h period compared with *U*. *pseudorotundata* (Supplemental Fig. S2). There were strong biomass accumulation (RGR) differences between *Ulva* strains with RGR values ranging from 0.09 mg.mg^−1^.d^−1^ to 0.37 mg.mg^−1^.d^−1^, and a CV of 28.8% (Supplemental Dataset 1). However, the species effect was not significant, with all species displaying similar RGR overall (*P* > 0.05; Fig. 3D). Furthermore, to investigate whether specific geographical locations contained more fast growing strains than other areas, we performed a principal component analysis on growth related traits for the 49 strains described below, and compared the results with their sampling locations. The analysis did not include sampling locations containing less than three stains, which excluded three strains located in two green tidal areas in Ireland. We found no evidence of clustering of strains based on sampling site (Supplemental Fig. S3), indicating that fast and slow growing strains coexist within the same habitats.

These results indicate the presence of strong inter and intraspecific genetic factors influencing the tissue expansion and biomass accumulation patterns across the studied *Ulva* strains.

### Physiological and Metabolic Factors Associated with Growth

**Figure 4.**
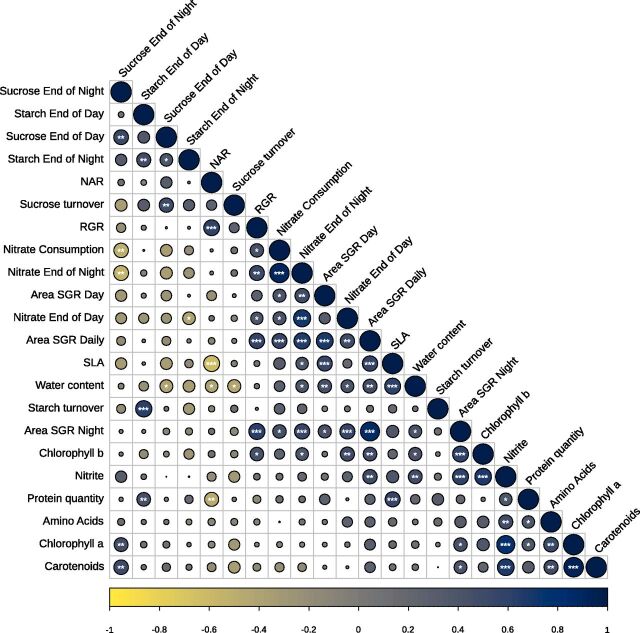
Spearman’s correlation matrix between growth and physiological/metabolic parameters. The color and size of the circles represent the correlation coefficient between pairwise comparisons. Asterisks indicate significance levels for each comparison, at FDR below 0.05 (*), 0.01 (**), and 0.001 (***); *n* = 49 strains. NET, net assimilation rate.

Several factors could influence the extensive strain growth differences observed in *Ulva*: (1) physiological/morphological factors such as water content, specific leaf area (SLA) or pigments, and/or (2) metabolic factors such as protein amounts and primary metabolites contents and/or diurnal variations. We, therefore, measured all of these traits for each investigated strain and generated a Spearman correlation matrix to unravel the relationships between each of those variables (Fig. 4; Supplemental Dataset 1).

#### (1) Physiological factors

We found that water content and SLA positively correlated with daily tissue expansion (Area SGR; R > 0.4). Moreover, strains with high amounts of chlorophyll b also displayed faster Area SGR during the night period and daily (24 h; R = 0.61 and 0.46, respectively). Chlorophyll a and carotenoid content only correlated with Area SGR during the night (R = 0.45 and 0.41, respectively). On the other hand, when growth was measured as biomass accumulation, RGR did not correlate with water content, SLA, chlorophyll a, or carotenoids, and only correlated with chlorophyll b content (R = 0.45). These results suggest that different physiological traits explain tissue expansion and biomass accumulation in *Ulva*. Indeed, we observed a strongly significant, albeit relatively low, correlation between daily and night Area SGRs and RGR between the 49 strains tested here (R = 0.57 and 0.63, respectively).

#### (2) Metabolic factors

Protein levels extensively varied between *Ulva* strains and species, ranging, from 40 mg.g DW^−1^ (*U*. *australis*) to 90 mg.g DW^−1^ (*U*. *laetevirens*), with a CV of 26.71% (Supplemental Fig. S4). There was no correlation between protein content and either tissue expansion or biomass accumulation; however, protein levels positively correlated with nitrite levels (R = 0.4). We found that nitrates accumulated in *Ulva* tissues during the night and were consumed during the day, with large variations among *Ulva* strains, from 0.26 to 6.52 mg NO_3_^−^-N.g DW^−1^ accumulated at the end of the night, CV of 48% (Supplemental Fig. S5). Nitrate quantity at the end of the night, end of the day, and its consumption during the day were all positively and significantly correlated with RGR and Area SGRs (Fig. 4; Supplemental Fig. S5). Thus, high nitrate accumulation in the night and consumption during daytime is associated with fast growth in *Ulva*, in both tissue expansion and biomass accumulation contexts. Carbohydrates (starch and sucrose) accumulated during the day and were consumed during the night (Supplemental Fig. S6), similar to the behavior of land plants (Geigenberger and Stitt, 2000; Gibon et al., 2009). Starch content was ∼50 times higher than sucrose at the end of the day; however, there was no significant correlation between their contents at the end of the day, or between starch turnover and tissue expansion or biomass accumulation (false discovery rate [FDR] > 0.05; Fig. 4).

### Evaluation of the Carbon Requirements for Night Growth and Identification of the Contributing Metabolites

When comparing the amount of starch and sucrose at the end of the day and at the end of the night, we found that almost all *Ulva* strains consume both carbohydrates during the night (Supplemental Fig. S6). However, sucrose contributed very little in contrast with starch. Interestingly, very large differences were observed among strains with 0 to 25 and −200 to 600 µmol equivalent glucose per g DW^−1^ for sucrose and starch, respectively. Some strains, such as TIE7 or VEN14, did not consume any starch or sucrose during the night period, despite their growth. Moreover, the rate of starch or sucrose degradation did not correlate with Area SGR at night (R = 0.13 and −0.05, respectively; FDR > 0.05). These results suggest the major involvement of another C source.

Hence, we estimated the amount of Carbon required for *Ulva* growth at night and compared the results with the amount of C available via the degradation of both starch and sucrose (Table 1; full table available in Supplemental Table S2, see “Materials and Methods” for details). On average, starch and sucrose degradation at night accounted for only as little as 35% of the C required, because, on average, 0.057 mg.mg DW^−1^ of C was required, whereas only 0.02 mg.mg DW^−1^ C was produced by starch and sucrose degradation at night. Given that another C source must therefore be present in *Ulva* to maintain the night growth, we determined the metabolites present in the strains by gas chromatography-mass spectrometry (GC-MS).

**Table 1.** Carbon from starch and sucrose turnover is not sufficient to sustain growth

Value	RGR (mg.mg d^−1^)	SGR Daily (%.d^−1^)	SGR Night (%.period^−1^)	SGR Night/SGR Daily (%)	RGR During Night (mg.mg night^−1^)	Ash Content (% DW)	Organic Matter Increase (mg.mg DW^−1^)	Carbon Required (mg.mg DW^−1^)	Carbon Used by Starch and Sucrose (mg.mg DW^−1^)	Carbon Missing (mg.mg DW^−1^)
Mean	0.209	13.473	8.636	65.450	0.137	23.072	0.105	0.057	0.020	0.037
sd	0.062	5.214	3.380	10.825	0.045	4.142	0.035	0.019	0.010	0.023

### Metabolites Determined by GC-MS as Biomarkers for Growth

**Figure 5.**
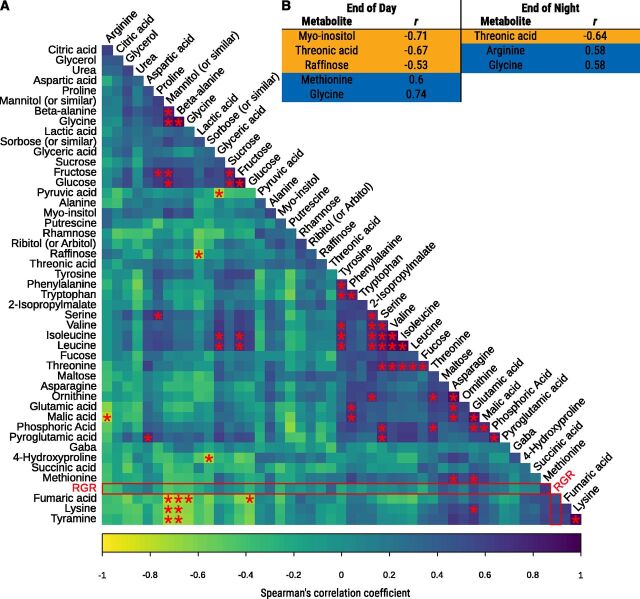
Correlations between the diurnal patterns of metabolite accumulation and growth. A, Spearman correlation matrix between end of day/end of night metabolite difference. RGR comparisons are indicated by a red rectangle. Asterisks represent significant correlations (q-value < 0.05, *n* = 16 strains). B, Metabolites significantly correlated with RGR at the end of day and end of night. Blue and orange indicate positive and negative correlations, respectively.

We investigated the metabolic profile of 16 other strains of *Ulva* spp. at the end of day and end of night by GC-MS, under a lower light intensity of 80 µmol m^−2^s^−1^, to place the strains under a moderate C limitation and then facilitate the identification of metabolites required for night growth. We identified 47 metabolites (organic acids, carbohydrates, and amino acids) from the GC-MS profile (Supplemental Dataset 2) and compared the metabolite levels at both timepoints with the biomass accumulation (RGR) of the 16 stains (Fig. 5B; Supplemental Fig. S7). We found six metabolites with significant (q-value < 0.05) correlations with RGR, at the end of day or end of night. Methionine and glycine were positively correlated with RGR at the end of day (R = 0.6, 0.74, respectively), whereas myo-inositol, threonic acid, and raffinose were negatively correlated (R = −0.71, 0.67, and −0.53, respectively). For end of night samples, glycine and arginine correlated positively with RGR (R = 0.58 for both), and threonic acid correlated negatively (R = −0.64; Fig. 5B; Supplemental Dataset 2). All the other metabolites failed to reach the significance threshold.

We then compared the accumulation or consumption of all the 47 metabolites during the night period (Supplemental Fig. S8) and found that 17 out of the 47 metabolites were consumed during the night (Log2 Fold change > 1; Supplemental Dataset 2). All these metabolites are thus putative candidates for an energy storage role. However, when we correlated RGR with the difference of the metabolite amount at the end of the night and the end of the day, no significant correlations could be observed (Fig. 5A). Hence, it is likely that none of the metabolites we determined have a major role as transient carbon/energy store for night growth and maintenance.

## DISCUSSION

### Development of a High Throughput Phenotyping Platform

In recent years, the computerization of plant growth monitoring has allowed for an increased throughput in the number of plant species/strains/ecotypes investigated for growth differences (Fahlgren et al., 2015; Fort et al., 2016). Although some setups can be expensive and/or complicated (Nagel et al., 2012; Sirault et al., 2013), the use of small and cheap computer systems, such as offered by Raspberry Pi computers and cameras (Minervini et al., 2017), together with a relatively low-level informatics pipeline, allow for the creation of cheap and easy-to-use phenotyping platforms. In addition, we show in this study, in agreement with the literature (Titlyanov et al., 1996; Gordillo et al., 2001; Ale et al., 2011; Kalita and Titlyanov, 2013), that the disc-based proxy systems are appropriate for detecting differences in growth performance between *Ulva* strains, because a strong correlation was found between thallus growth and disc growth (Fig. 1C). This finding is of importance because it demonstrates that disc-based screenings are likely to identify strains that could lead to higher biomass yield in farming systems. Although wounding of tissue was shown previously to trigger sporulation in *Ulva rigida* (Gao et al., 2017), no major sporulation events were observed in our system, possibly because the ∼12-mm disc diameter used was sufficiently large to avoid excessive wounding. Indeed, Gao et al., 2017 found little to no sporulation occurring in discs of >10 mm in diameter. Overall, the phenotyping system described in this study represents a high throughput, cheap, and reliable (Fig. 1) method for *Ulva* strain selection, and could also be used to precisely investigate *Ulva* response to various environmental conditions.

### Extensive Natural Variation in Growth among *Ulva* spp. Strains, and Implications for Aquaculture

We found that *Ulva* strains show a large degree of variation in growth, whether based on tissue expansion (Area SGRs) or biomass accumulation (RGR). Indeed, a 28% and 35% CV were observed for RGR and Area SGR, respectively (Fig. 3). Variations in RGR reported among Arabidopsis (*Arabidopsis thaliana*) accessions are usually much lower with, for example, a CV of ∼8% observed for area SGR among >300 accessions of Arabidopsis (Bac-Molenaar et al., 2015), or 13% for RGR (Cross et al., 2006). Hence, careful strain and species selection could lead to major increases in *Ulva* biomass yield for in-land (Bolton et al., 2009; Lawton et al., 2013) or off-shore *Ulva* farming systems (Korzen et al., 2016; Lehahn et al., 2016). In addition, although the species effect on RGR was not significant, larger sampling sizes for each species might detect overall differences. Indeed, the difference in RGR between *U. gigantea* and *U. pseudorotundata* strains, although not significant (*P* = 0.059), was still ∼ 0.07 mg.mg^−1^.d^−1^, and over a growth period of several months could lead to a difference in large biomass yield. Nonetheless, the extensive growth variation observed in our dataset indicates that prior selection of the best strains (regardless of species) for specific uses is of tremendous importance for aquaculture. Such strain selection has, to date, been mostly restricted to *Porphyra* spp., *Laminaria* spp., and *Saccharina japonica* (Robinson et al., 2013). Notably, once suitable strains are selected, the creation of hybrids could also increase the subsequent yield of the crops (Li et al., 2008) by harnessing the heterotic potential of hybrids (McKeown et al., 2013).

Importantly, we found no correlation between protein/starch amounts and growth, indicating that strain selection based on protein and/or starch content for food, feed, and bioenergy is likely possible without a trade-off in biomass yields. However, the conditions used in this study are close to optimal growth conditions, with light and nutrients likely being in excess. Protein/starch amount could become more limiting for growth under carbon or nutrient-limiting conditions, and new strain selection should be performed using environmental conditions tailored for specific aquaculture systems. The wide range of nitrate accumulation during the night and its usage during the daytime among the strains indicate that strain selection could also be used for nutrient removal in waste-water integrated multitrophic aquaculture systems (Ben-Ari et al., 2014; Rabiei et al., 2014; Amosu et al., 2016). Moreover, because strains with high nitrate accumulation are also among the strains that grew the fastest, there is also a clear economic potential for combining nutrient removal and subsequent use of this biomass for food/feed/bioenergy.

Strikingly, our recent study assessing the genetic diversity of *Ulva* spp. using genotyping-by-sequencing showed a relatively small, but detectable, genetic difference between *Ulva* strains (Fort et al., 2018). The presence of large growth and metabolic variation within *Ulva* despite low overall genetic variation points toward the presence of a relatively small number of genes possessing a strong influence on these traits. We hypothesize that the application of a genome wide association study in *Ulva* using our high throughput phenotyping platform is likely to yield important genetic markers and gene candidates for future strain selection.

### Biomass Accumulation Is Explained by Both Thallus Area Expansion and Thickness

Growth measured as tissue expansion and biomass accumulation are strongly and significantly correlated, but with a relatively low correlation coefficient (R = 0.56; Fig. 4). Disc size increase and biomass accumulation do not necessarily have to be strongly correlated. Indeed, strains could vary in dry weight content and thickness of their thalli; thus an identical increase in thallus area could lead to different biomass accumulation. The slight discrepancy between tissue expansion and biomass accumulation we observed could be explained by the differences in water content and SLA observed among the 49 tested strains. Indeed, SLA correlated negatively with net assimilation rate, which itself correlates with RGR, demonstrating that tissue expansion and biomass accumulation are not completely connected in laminar *Ulva* spp.

### Most of *Ulva* Growth Is Performed at Night

The *Ulva* tissue expansion (Area SGR) pattern observed in this study is particularly striking, because we observed that *Ulva* mostly grows during night time, which is the opposite of all land plants whose diurnal growth pattern has been described (Ben-Haj-Salah and Tardieu, 1995; Berman and DeJong, 1997; Walter et al., 2009; Sulpice et al., 2014). Our results confirm those from Titlyanov et al., 1996, who showed, for a single strain of *U. pseudocurvata*, that the pattern of growth and cell division peaked at the end of night and at the end of day, respectively. Such a peculiar growth pattern could be explained by an adaptation to avoid growth during the day, where the mutagenic effects of UV radiation from sunlight could lead to a decreased fitness in the wild. However, we would expect such limitations to also exist among land plants, and to our knowledge, all grow as fast or slower at night than during the daytime (Walter et al., 2009; Poiré et al., 2010). Moreover, we found that *U. gigantea* strains grow similarly during the day and night, further indicating that a simple avoidance of UV exposure is unlikely to explain the growth pattern of *Ulva*, since it appears species specific.

Another explanation would be that cell division and expansion need to be tightly synchronized so that all peptides and other building blocks required to produce new structures are produced synchronously. This process is largely under diurnal control (Flis et al., 2016), with the circadian clock playing a major role, at both the transcriptional and translational levels (Missra et al., 2015). For example, the expression of genes involved in photosynthesis mostly peaks toward the end of the night, thus facilitating the timely stoichiometric production of the subunits of important photosynthetic complexes. Indeed, many of the complexes involved in photosynthesis are formed of numerous proteins, and their assembly requires all proteins to be present in the right amount, at the right time during the diurnal cycle. Moreover, such regulation would allow time for the accumulation of enough metabolites to sustain the complete formation of the protein/cell structures required for growth. Variations in the diurnal time (ZT) of cell division have been observed in cyanobacteria, microalgae, and macroalgae. Some species of cyanobacteria divide during the day (Mori et al., 1996; Asato, 2003), whereas others divide during the night (Campbell and Carpenter, 1986). In unicellular algae, however, cells divide during the night (Sweeney and Woodland, 1958; Goto and Johnson, 1995; León-Saiki et al., 2018), and the brown algae *Pterygophora californica* grows during the day (Lüning, 1994). Those studies highlight large variations in the growth patterns among autotrophic aquatic organisms. Thus, the evolutionary, adaptative, and/or metabolic significance behind the growth pattern of *Ulva* remains to be investigated. Moreover, the diurnal differences in growth observed between *Ulva* species could be of importance for aquaculture, because it likely indicates variable responses to photoperiods.

### Nitrate Assimilation as a Biomarker for Growth

Nitrate is of tremendous importance in the environment and in aquaculture. Indeed, nitrification of seawater has been associated with the occurrence of green tides across the globe (Ye et al., 2011), and nutrient removal represents a significant challenge in coastal and land-based fish aquaculture (Crab et al., 2007). One such solution is to use macroalgae such as *Ulva* to capture the nitrogen released by the farming systems and use the subsequent biomass for feed (Troell et al., 2009; Shpigel et al., 2017). Here, we found that *Ulva* is indeed capable of assimilating large quantities of dissolved nitrogen and using this assimilated nitrate for growth. Importantly, we observed a significant correlation between growth (biomass accumulation or tissue expansion based) and nitrate assimilation (Fig. 4), indicating that selection based on nitrate assimilation is likely to yield fast-growing strains. Indeed, the strong differences in nitrate accumulation during the night between strains could be due to strain and species-specific nitrate uptake efficiency (Supplemental Fig. S7), which represents a significant biomarker for strain selection. Moreover, ∼80% of the nitrate assimilated during the night in *Ulva* is consumed during the day, and such consumption is also correlated with growth. This pattern can be explained by the light-dependent activation of nitrate reductase through posttranscriptional regulation and dark-dependent degradation (Huber et al., 1996; Weiner and Kaiser, 1999; Esteves-Ferreira et al., 2018), as well as the next step, the reduction of nitrite to ammonia by nitrite reductase, which occurs in the chloroplasts and requires energy from the light reactions of photosynthesis. Overall, these results indicate that nitrate metabolism could play a central role in future breeding efforts on this species.

### Metabolites Associated with Growth in *Ulva*

Previous studies have shown that *Ulva* produces large quantities of starch (Korzen et al., 2016), and it was hypothesized that starch, together with ulvans, represent the main carbohydrate reserves used for energy storage (Bruhn et al., 2011). Although long-term storage could indeed be assured, in part, by starch, the diurnal pattern of starch accumulation and degradation in *Ulva* had not yet been investigated, nor were night growth rates. Here, we used the discs collected at the end of day and end of night, together with growth rate data, to assess whether starch (or other soluble metabolites detected by GC-MS) could act as primary photoassimilates to sustain night growth, such as in Arabidopsis (Sulpice et al., 2009, 2014). The carbon released by starch turnover was not sufficient to account for the carbon required for growth. Similarly, sucrose poorly contributed to the C requirements for night growth, and both compounds accounted for only ∼35% of the requirements (Table 1), thus suggesting the presence of another carbon source to sustain night growth. We then queried whether 47 other soluble metabolites detected by GC-MS could play a possible role in supplying carbon for night growth. Given the fact that our data are only semiquantitative, we searched for correlations between RGR and the relative amounts of metabolites.

In Arabidopsis, it was previously reported that glycine, threonic acid, and alanine levels at the end of day negatively correlate with biomass accumulation (Sulpice et al., 2009). In *Ulva*, however, glycine was found to be positively correlated with growth, whereas myo-inositol, arginine, raffinose, and methionine did not correlate with biomass accumulation in Arabidopsis. Threonic acid was the only metabolite negatively correlated with growth in both Arabidopsis and *Ulva*, highlighting important metabolic differences between the two organisms. Although each of those metabolites could have a role in modulating the growth of *Ulva*, none of them showed a pattern of accumulation during daylight and consumption during night correlating with growth (Fig. 5A; Supplemental Fig. S8). This makes them unlikely candidates for a reserve role in sustaining the high growth of *Ulva* during the night (Fig. 5B). To date, metabolic analysis of macroalgae species within a diurnal cycle was restricted to *Ectocarpus*, and mannitol was found to be the likely transient carbon source (Gravot et al., 2010). In *Ulva*, however, mannitol content appears stable between night and day (Supplemental Fig. S8; Supplemental Dataset 2), highlighting the important metabolic differences between macroalgae species, as demonstrated by a recent study (Belghit et al., 2017). Hence, the metabolite responsible for *Ulva* night growth remains elusive. This metabolite might not have been identified among the GC-MS profiles or may be an ethanol-insoluble metabolite, such as ulvan. However, whether such a complex sulfated polysaccharide, a polymer of rhamnose, xylose, glucose, galactose, glucuronic acid and iduronic acid (Robic et al., 2009), can play the role of short-term storage of carbon for sustaining night growth remains to be investigated. Identifying the main transient carbon source in *Ulva* represents an important future area of investigation, because it would represent a promising biomarker for growth in breeding programs.

### Ecophysiological Implications for *Ulva* Blooms

*Ulva* blooms are seasonal phenomena where a large volume of coastal environment is taken over by *Ulva* biomass in a short period of time (Liu et al., 2010; Keesing et al., 2011). Among the abiotic factors that could trigger such events, the eutrophication of the estuarine environment can be considered as the main factor leading to the increasing number of blooms recorded worldwide (Smetacek and Zingone, 2013; Whitehouse and Lapointe, 2015). Here, we show a link between the nitrogen assimilation of *Ulva* strains and their growth, strengthening the causality between eutrophication and the occurrence of *Ulva* blooms. Furthermore, *Ulva* blooms are rarely restricted to a single *Ulva* species (Merceron et al., 2007; Chávez-Sánchez et al., 2018; Bermejo et al., 2019), indicating that several *Ulva* species have the growth capability to overtake large volumes of shore. We found in this study that, although a large variation in biomass accumulation (RGR) is present among *Ulva* strains, no interspecific growth differences were observed between the *Ulva* species investigated here (Fig. 3D). However, when tissue expansion (Area SGR) is considered, we found significant differences between *Ulva* species (Supplemental Fig. S2), indicating a possibly variable potential for *Ulva* species to take over coastal areas when blooming. During such green tide events, *Ulva* strains that expand the fastest would be expected to out-compete strains with lower Area SGRs, regardless of their biomass accumulation potential. Thus, green tidal areas might be enriched for *Ulva* strains with specific physiological characteristics, such as high-water content, SLA, and Area SGRs. Further studies of green tide versus nongreen tides strains of *Ulva* might shed light on the putative genetic basis of the occurrence of green tide worldwide. In the case of nongreen tide areas, high Area SGR and fast expansion, or even high RGR are likely less critical for survival, as we found within the same geographical areas large variation in growth characteristics among the strains we collected (Supplemental Fig. S3), which didn’t include green tide areas.

Taken together, this study demonstrates a large natural variation in the growth and metabolite content of strains and species of laminar *Ulva*, indicating that careful selection of strains before use in aquaculture could lead to significant increases in biomass, protein, and/or carbohydrate content, depending on the end-product application of the *Ulva* biomass. Notably, we show that nitrate content in *Ulva* thallus could be used as a biomarker for growth, as could the abundance of six amino acids. Future work will focus on precisely deciphering the primary metabolism of this group of species, specifically on identifying its short-term carbon storage compound(s), and use of the phenotyping platform to expand the number of *Ulva* strains characterized in order to allow for genome-wide association mapping on several relevant traits for aquaculture. Hence, the data presented here represent an important stepping stone toward the understanding of growth of this environmentally and commercially relevant taxa and can be used to direct future breeding efforts toward higher yielding strains.

## MATERIALS AND METHODS

### Algae Materials and Growth Conditions

Laminar *Ulva* spp. Strains were collected from intertidal and subtidal locations throughout Ireland and the Netherlands (Supplemental Table S1). The samples were placed in resealable bags onsite, filled with seawater, and kept in a chilled container. Upon arrival in the laboratory, the tissue samples were wiped with paper tissue to remove possible epiphytes. The holdfast and its surrounding area were removed to ensure better homogeneity of the tissue, and the remaining thalli were placed in 250 mL glass jars filled with media containing artificial seawater (34 g L^-1^; Red Sea Coral Pro) and 1X Cell-HI F2P vitamins and nutrients (Varicon aqua), at constant temperature (15°C) under fluorescent light (Osram T5 tubes, ∼80 µmol m^−2^s^−1^ Photosynthetically active radiation). All samples were allowed to grow under these conditions for at least 2 weeks (> 6 months for some of them). With a RGR of ∼0.16 mg.mg^−1^.d^−1^ for thallus growth (Fig. 1C), we expect most of the tissue to have been generated in the lab under controlled conditions within the acclimation period. To ensure total acclimation of all strains, before growth monitoring, the *Ulva* strains were further acclimated under the growth phenotyping platform’s LED lights for at least one more week. When the media where a strain was growing appeared to be contaminated by microalgae, this strain was excluded from the screen.

### High throughput Platform for Growth Monitoring in Laminar *Ulva* spp.

A custom-made phenotyping platform was designed to simultaneously monitor the growth of dozens of individual laminar *Ulva* spp. strains by means of 2D imaging. When cut into a disc, laminar *Ulva* grow in all directions from the cut piece of tissue, allowing growth to be measured by comparing the changes in disc area over time. A similar imaging approach had been previously used to monitor growth in *Ulva* spp. thalli under different environmental conditions (Ale et al., 2011; Lawton et al., 2013). To increase efficiency and monitor day and night growth of hundreds of *Ulva* samples, we used a network of low-cost, Raspberry Pi computers, each with their own Raspberry Pi camera (Radionics). The computers were programmed to take pictures of the biomass every 5 min for the duration of the growth experiment. The advantage of such equipment is their low cost and the possibility to link and remote-control all computers on the same network, compared with the use of expensive digital single-lens reflex cameras. Once the still images of the samples were captured, a modified segmentation script (Fahlgren et al., 2015) was automatically applied on the images to remove noise from the images, leaving only the *Ulva* spp. discs. All the images belonging to a single 30-min segment were merged using ImageMagick (https://www.imagemagick.org) to reduce possible noise due to water movement within the tanks by evaluating the sequence mean and generating a consensus image for the 30-min segment. Finally, ImageJ (https://imagej.nih.gov/ij/) was used to stack the segmented images and calculate the increase in disc areas over the course of the experiment (Fig. 1A).

Together with the image capture and analysis pipeline, the setup in which to grow the *Ulva* samples also had to be designed (Supplemental Fig. S9). To effectively monitor the growth of hundreds of *Ulva* discs automatically, we placed the discs in 25L aquarium tanks containing 5L of artificial seawater media as described above. The use of artificial seawater and subsequent addition of the missing macronutrients were deemed essential to ensure a constant composition of the growth media over time. To maintain the *Ulva* discs flat and immobile in the tanks, individual discs were placed at the bottom of 6-well plates (#83.3920.500, Sarstedt) maintained in position by three-dimensional printed O-rings (in PLA plastic, using a MakerBot Replicator 2 printer, MarkerBot), and a white nylon mesh (Hickeys Fabrics) of 3 mm grid size glued to the O-rings with clear TEC7 silicone (Woodies). The setup allowed 2D growth in the wells, while avoiding folding of the biomass on itself, disc escape from the wells, and ensuring that the discs were always perpendicular to the cameras. To ensure efficient circulation of the nutrients and allow for water movement within the tanks, small aquarium pumps were added. One camera was placed on the top of each tank, and with this setup, 36 to 42 individual discs could be monitored for growth in a single aquarium tank. The tanks were placed in a constant temperature room (15°C ± 1°C), with the discs illuminated at 200 µmol m^−2^s^−1^ photosynthetically active radiation with Spectron T8 LED 1.5 GB tubes (HydroGarden), with a photoperiod of 12 h of light and 12 h of darkness. The light intensity and quality of the LED tubes was selected to best reflect possible coastal or aquaculture setups (Merceron et al., 2007; Mata et al., 2010; Zhu et al., 2014). The positions of the discs from each strain were randomized across the eight tanks of the phenotyping platform to ensure a homogenous dataset with no blocking effects. For each strain, we used three discs per replicate and three replicates per time point (end of day and end of night). At the end of the experiment, for metabolic analyses, the discs of each strain were harvested randomly among the tanks at both time points and immediately flash frozen in liquid nitrogen. The fresh weight of the frozen discs was then measured, and the discs were subsequently freeze-dried for long-term conservation and metabolic analysis.

To compare the growth of *Ulva* thalli versus the growth of discs obtained from the same strain, we have grown thalli of *Ulva* individuals in the exact same conditions as those used under the phenotyping platform. The *Ulva* samples were placed in plastic jars, pierced on each side and with their lid removed and replaced by nylon mesh, to allow for easy water flow. The fresh weight of the *Ulva* samples was measured before and after 1 week of growth. Next, discs from the same thalli were cut and grown in the phenotyping platform as described above.

### Growth Parameter Analysis

Growth was estimated based on several parameters. The 2D imaging pipeline allowed for a precise measurement of disc area increase over the course of the experiment. The data generated were used to measure Area SGR in percent increase per given period (nighttime, daytime, and/or a 24-h period), using the following calculation:



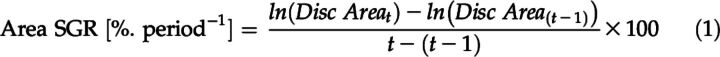

Area SGR was measured daily using 2D still imaging over the course of the experiments (6 d), and for any given disc the final area SGR was computed as the mean of six area SGRs.

Second, growth was measured as the biomass (dry weight) accumulated per unit of dry weight over time in the form of RGR, in milligrams.milligrams^−1^.day^−1^.



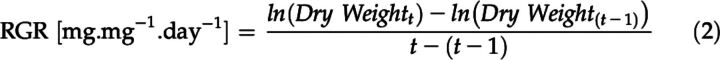

RGR was also subdivided into two separate components, SLA, the ratio of thallus area to dry biomass, and NAR, which represents the dry biomass accumulation per thallus area per day:



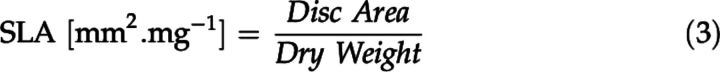





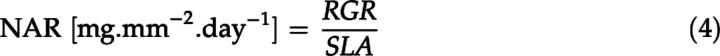



### DNA Extraction, Sanger Sequencing, and Phylogenetic Analysis

DNA extraction was performed using magnetic beads as described by (Fort et al., 2018). For Sanger sequencing, 1 µL of extracted DNA was amplified by PCR using primers specific to *RbcL* (Heesch et al., 2009) and *tufA* (Saunders and Kucera, 2010). PCR amplicons were sent to Laboratory of the Government Chemist genomics GmbH for Sanger sequencing. Sequencing traces for all samples were aligned using Multiple Sequence Comparison by Log-Expectation (Edgar, 2004), and the aligned sequences for both RbcL and tufA were concatenated using Molecular Evolutionary Genetics Analysis. Next, the final alignment was trimmed with trimAl (Capella-Gutiérrez et al., 2009) using the -automated1 argument to avoid spurious gaps in the alignment. Finally, species delimitation was performed using a GMYC model (Pons et al., 2006; Fujisawa and Barraclough, 2013): the concatenated sequences for *RbcL* and *tufA* of the *Ulva* samples and a set of most likely candidate species sequences (determined by BLAST) were analyzed using the Molecular Evolutionary Genetics Analysis software under a Yule model (Bouckaert et al., 2014). The resulting tree and species identification were determined using the splits package in R (Fujisawa and Barraclough, 2013), and visualized using FigTree (http://tree.bio.ed.ac.uk/software/figtree/).

### Metabolic Analyses

Freeze-dried discs were ground into powder using a TissueLyser II bead mill (Qiagen), and ∼5 mg aliquots were placed in screw caps tubes for metabolite extractions. Soluble metabolites were extracted using hot ethanolic extraction (Esteves-Ferreira et al., 2017). The resulting supernatant was used for chlorophyll, sugar, and nitrate analyses, whereas the pellet was used to determine protein and starch content. Soluble sugars were measured by an enzymatic reaction method (Stitt et al., 1989; Fernie et al., 2001), nitrate/nitrite content as per the Griess method (Tsikas, 2007), chlorophyll/carotenoid content according to Porra et al., 1989, and amino acids using ninhydrin (Yemm et al., 1955). Proteins were determined according to Lowry et al., 1951. Because ulvans, a primary component of *Ulva* biomass, could interfere with the Lowry method due to the copper binding properties of ulvans, we spiked *Ulva* extracts with bovine serum albumin to estimate percentages of recovery. Recovery rates were around 100% for all spiked amounts of bovine serum albumin, indicating that this protein detection method is appropriate (Supplemental Fig. S10). Starch was determined according to Hendriks et al., 2003; Smith and Zeeman, 2006 by measuring the amount of glucose released after enzymatic starch degradation. Starch, sucrose, and nitrate turnover between day and night were measured by calculating the difference in those metabolite amounts at the end of the day and at the end of the night. All assays were performed using 96-well plates. To control for potential inter plate variations during metabolite assays, two aliquots issued from a large pool of *Ulva* biomass were added to all the assay plates. Ash content in *Ulva* spp. tissue samples was measured by combusting the ground freeze-dried disc samples at 550°C in a furnace for 12 h.

To conduct GC-MS analysis, ∼5 mg of freeze-dried powdered dry weight was extracted using a methanol:chloroform:H_2_0 10:3:1 (v/v/v) solution with 1 µg/mL of palatinose (Sigma P2007), with two to three replicates per strain and time point (end of day and end of night). The samples were extracted by continuous shaking for 10 min at 4°C. The extracts were then centrifuged at 4°C for 2 min at 14,000 rpm, and 500 µL of supernatant was dried in a rotary vacuum at 30°C. The residue was derivatized for 120 min at 37°C (in 40 µL of 20 mg mL^−1^ methoxyamine hydrochloride in pyridine) followed by a 30-min treatment at 37°C with 70 µL of *N*-Methyl-*N*-(trimethylsilyl)trifluoroacetamide. An autosampler Gerstel Multi Purpose system was used to inject the samples into a chromatograph coupled to a time-of-flight mass spectrometer (GC-MS) system (Leco Pegasus HT TOF-MS). Helium was used as the carrier gas at a constant flow rate of 2 ml/s, and gas chromatography was performed on a 30-m DB-35 column. The injection temperature was 230°C, and the transfer line and ion source were set to 250°C. The initial temperature of the oven (85°C) increased at a rate of 15°C/min up to a final temperature of 360°C. After a solvent delay of 180 s, mass spectra were recorded at 20 scans s^−1^ with *m/z* 70-600 scanning range. Chromatograms and mass spectra were evaluated by using Chroma TOF 4.5 (Leco) and TagFinder 4.2 software. GC-MS peaks were normalized with the dry weight biomass used for the given sample.

### Carbon Requirement Estimations

The amount of carbon (C) required for growth was estimated using the following parameters:

We estimated the proportion of biomass increase during the night (because the water content between end of day and end of night samples is similar [Student’s *t* test, *P* = 0.1]; Supplemental Fig. S11]; we considered the night Area SGR to produce as much biomass as the day Area SGR per percentage of tissue expansion) using the following formula:

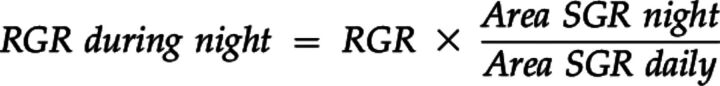

We considered the ash content of the samples (only 35 of the 49 strains were used here, since a minimum of 15 mg DW was used for ash determination), and calculated the amount of organic matter increase during the night using the following formula:

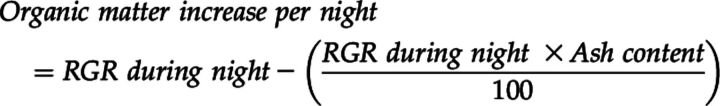

We considered *Ulva* biomass to contain ∼ 30% C (van der Wal et al., 2013) and a growth respiration factor of 1.39 to account for the amount of C required to produce 1 g of structural dry matter (Vries et al., 1979), to calculate the amount of C required to sustain night growth using the formula:

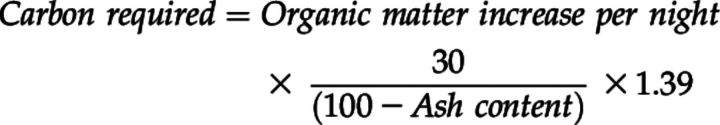

we calculated the amount of C released by starch and sucrose degradation at night by multiplying the amount of starch and sucrose degraded at night with the atomic mass of carbon and the number of carbon atoms in a glucose molecule.

### Data Analysis

All statistical analyses were performed using R (R Core Team, 2018). Correlation matrixes between growth, morphological, and metabolic traits were computed using the Hmisc package (Harrell and Dupont, 2008), and visualized using the Corrplot (Wei and Wei, 2017) package, from the mean data for each strain. To limit the identification of false positives, q-values were estimated for each Spearman’s pairwise test of the correlation matrixes, and only q-values with FDR < 0.05 were deemed significant. Computations of q-values and FDRs were performed using the qvalue package (Dabney et al., 2010). For GC-MS data comparison, we generated a correlation matrix between metabolite amount at the end of day or end of night and RGR, generated and analyzed using the Hmisc and qvalue packages, together with the difference between each metabolite amount at the end of day/end of night and RGR.

Statistical differences among *Ulva* species were tested by nested ANOVA using a linear mixed effect model, considering species as a fixed effect and strains within a species as random effect. The model was fitted using the lme4 package in R (Bates et al., 2015). The least squares means differences between species obtained from the linear model are compared using the lmerTest package in R (Kuznetsova et al., 2017) and the resulting p-values adjusted using the Tukey method.

Graphics were also generated in R (R Core Team, 2018) using the ggplot2 (Wickham, 2016) package and heatmaps using the gplots package (Warnes et al., 2016). Colors originate from the viridis package (Garnier, 2017).

### Supplemental Data

The following supplemental materials are available:

**Supplemental Figure S1.** RGR of *Ulva* discs following three acclimation periods under moderate intensity LED lights.
**Supplemental Figure S2.** Comparison of Area SGRs between *Ulva* species.
**Supplemental Figure S3.** PCA of growth related traits and geographical origin of the strains used in this study.
**Supplemental Figure S4.** Protein quantity in *Ulva* strains.
**Supplemental Figure S5.** Nitrate accumulation in *Ulva* tissue.
**Supplemental Figure S6.** Suc and starch concentration in *Ulva* tissues at the end of day and end of night.
**Supplemental Figure S7.** Spearman correlation matrix between metabolites at the end of day and end of night identified by GC-MS.
**Supplemental Figure S8.** Log2 Fold change between the amount of each metabolite at the end of the day and the end of the night.
**Supplemental Figure S9.** Design of the phenotyping platform.
**Supplemental Figure S10.** The Lowry method is appropriate for protein levels determination in the ethanol-insoluble fraction of *Ulva* tissue.
**Supplemental Figure S11.** Water content of *Ulva* disks at the end of day and end of night.
**Supplemental Table S1.** List of the 49 strains used in this study for growth analysis, with species identification and GPS coordinates.
**Supplemental Table S2.** Missing carbon calculations for 35 strains.
**Supplemental Dataset 1.** Mean, CV and s.d of the traits for the 49 strains and their correlation coefficients.
**Supplemental Dataset 2.** GC-MS dataset and correlations.

## Supplementary Material

Supplementary Data
