# Supplementary material for: Extensive Variations in Diurnal Growth Patterns and Metabolism Among Ulva spp. Strains
Source: Plant Physiol. 2019 Feb 12;180(1):109–23. doi: 10.1104/pp.18.01513 (PMC6501106; doi:10.1104/pp.18.01513)
Supplement: Supplementary Data [file plphys_v180_1_109_s1.zip › PP2018-RA-01513R1_Supplemental_Figures_1_11.pdf]

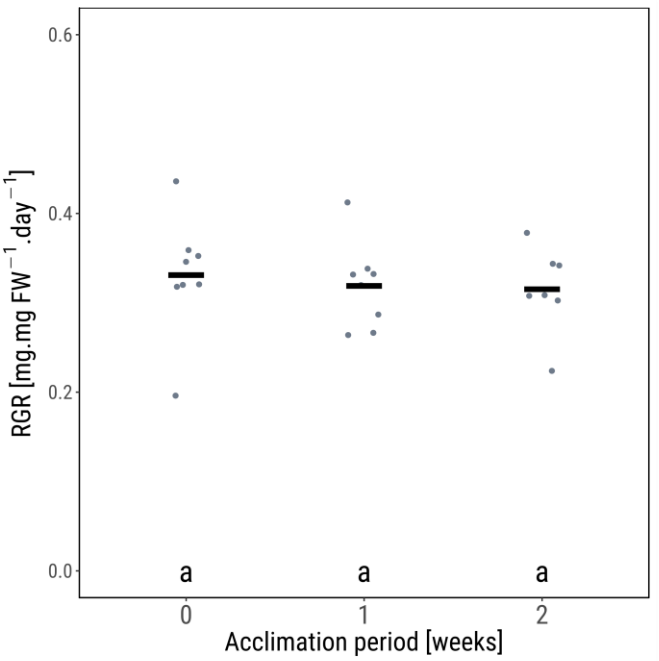

Supplemental Figure S1: RGR of *Ulva* discs following three acclimation periods under moderate intensity LED lights. Each disc's RGR is shown by a dot, horizontal bars represent the mean ($n = 8$ discs). Letter represent significance groups (One way ANOVA, $p < 0.05$).

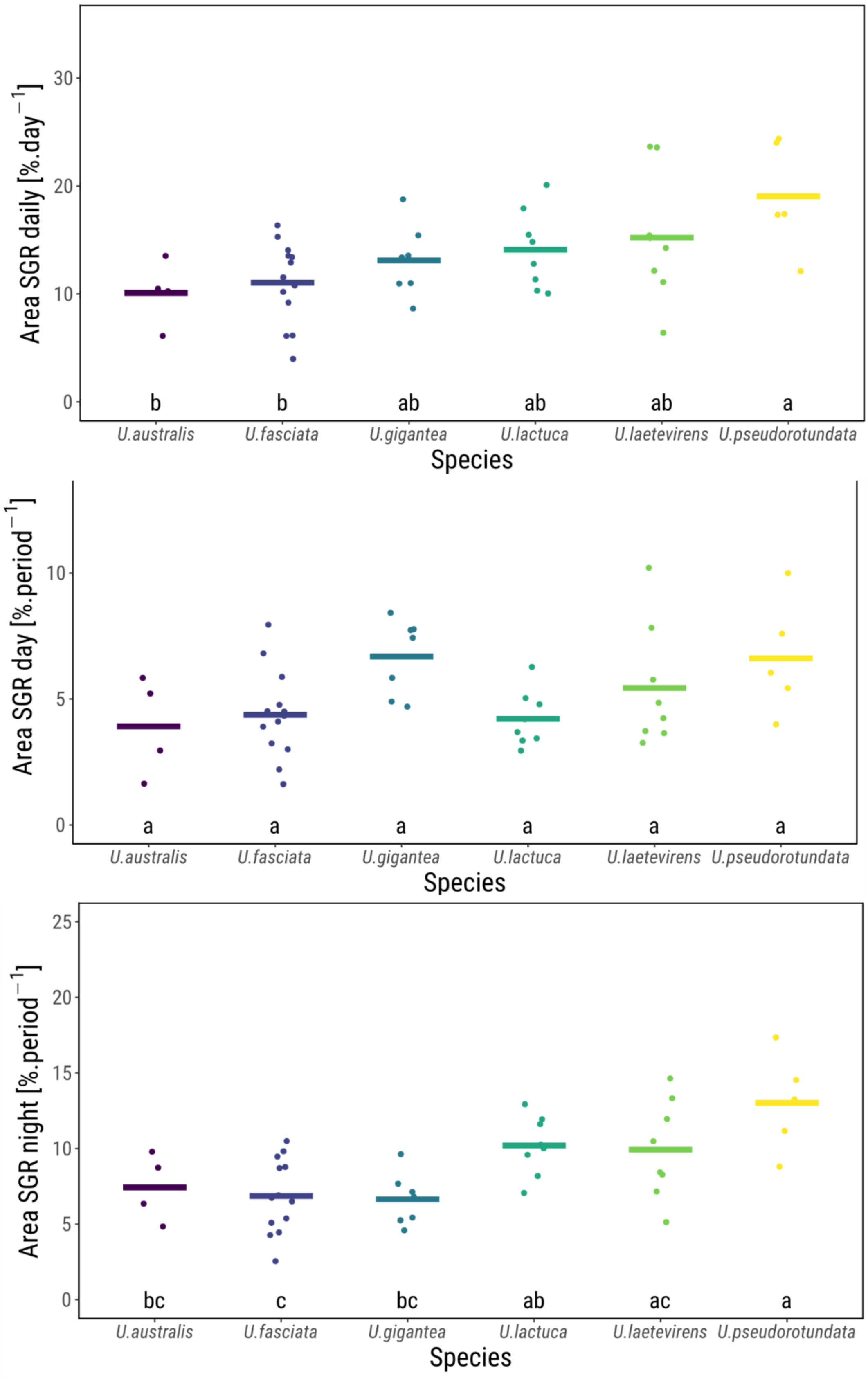

Supplemental Figure S2: Comparison of Area SGRs between *Ulva* species. Average Area SGR per species for daily (top), day (middle) and night (bottom). Dots represent the mean per strain, with horizontal line representing the mean per species. Letters represent significance groups (One way ANOVA, $p < 0.05$).

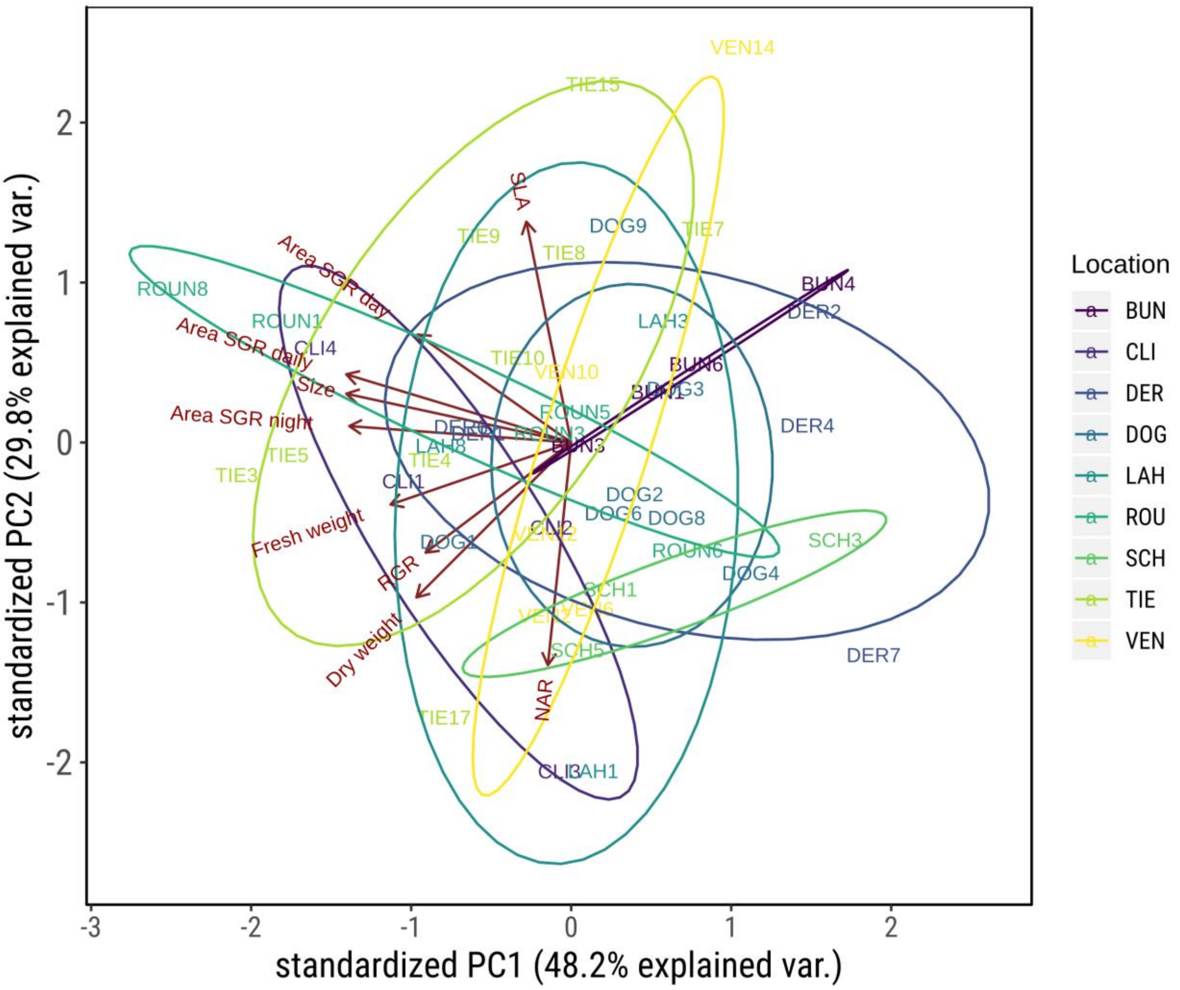

Supplemental Figure S3: PCA of growth related traits and geographical origin of the strains used in this study. Principal Component Analysis (PCA) between growth related traits obtained using the phenotyping platform. Colours represent the sampling location (see Supplemental Table S1), and only locations with more than two strains were used in this analysis.

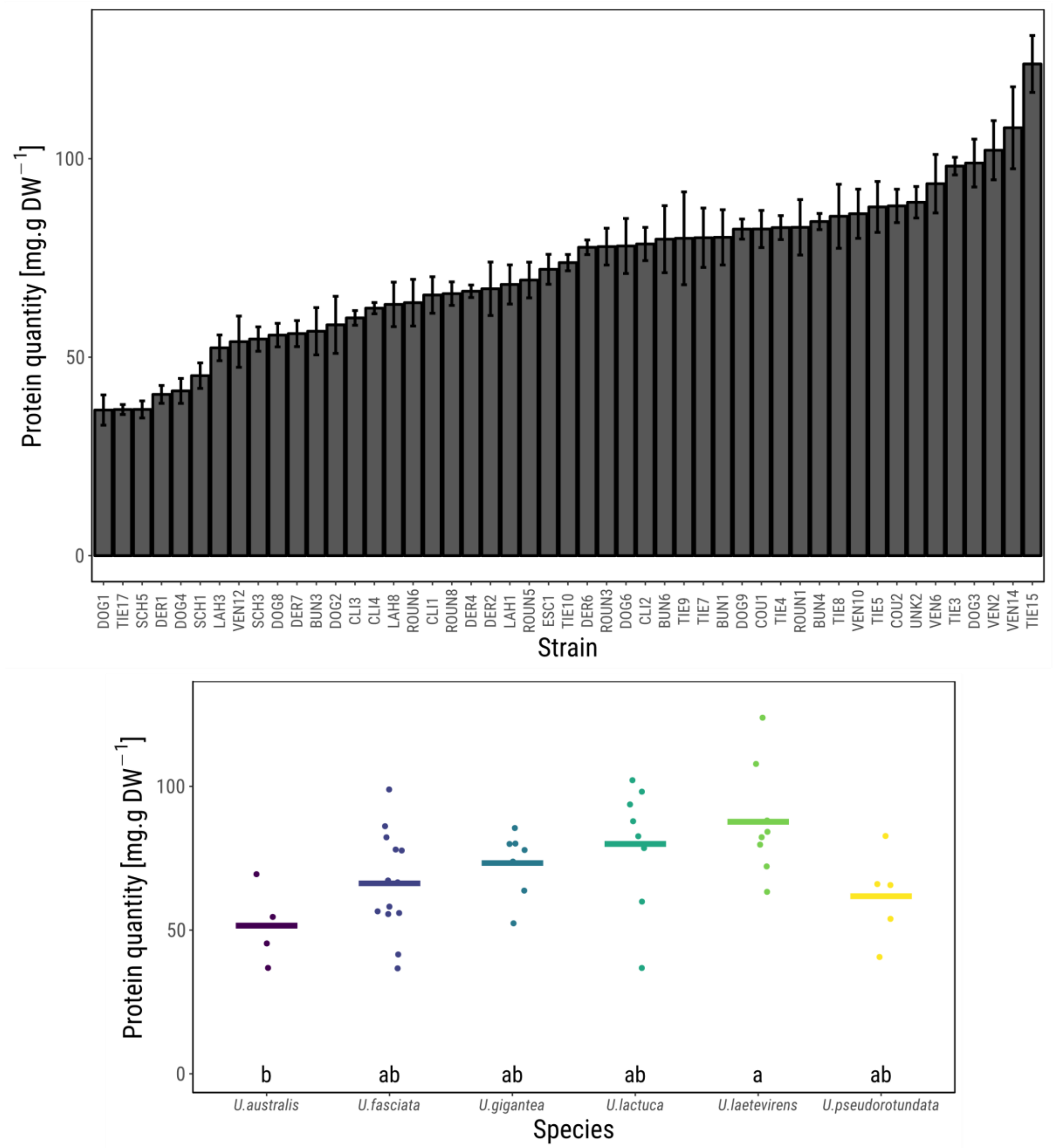

Supplemental Figure S4: Protein quantity in *Ulva* strains. Top: Protein concentration in each *Ulva* strain. Bars represent the mean ± s.e.m, n = 6 pools of three discs each per strain. Bottom: Average protein concentration for each *Ulva* strains (dots), separated by species. The horizontal line represents the mean per species. Letters indicate significance groups (Nested ANOVA, $p < 0.05$)

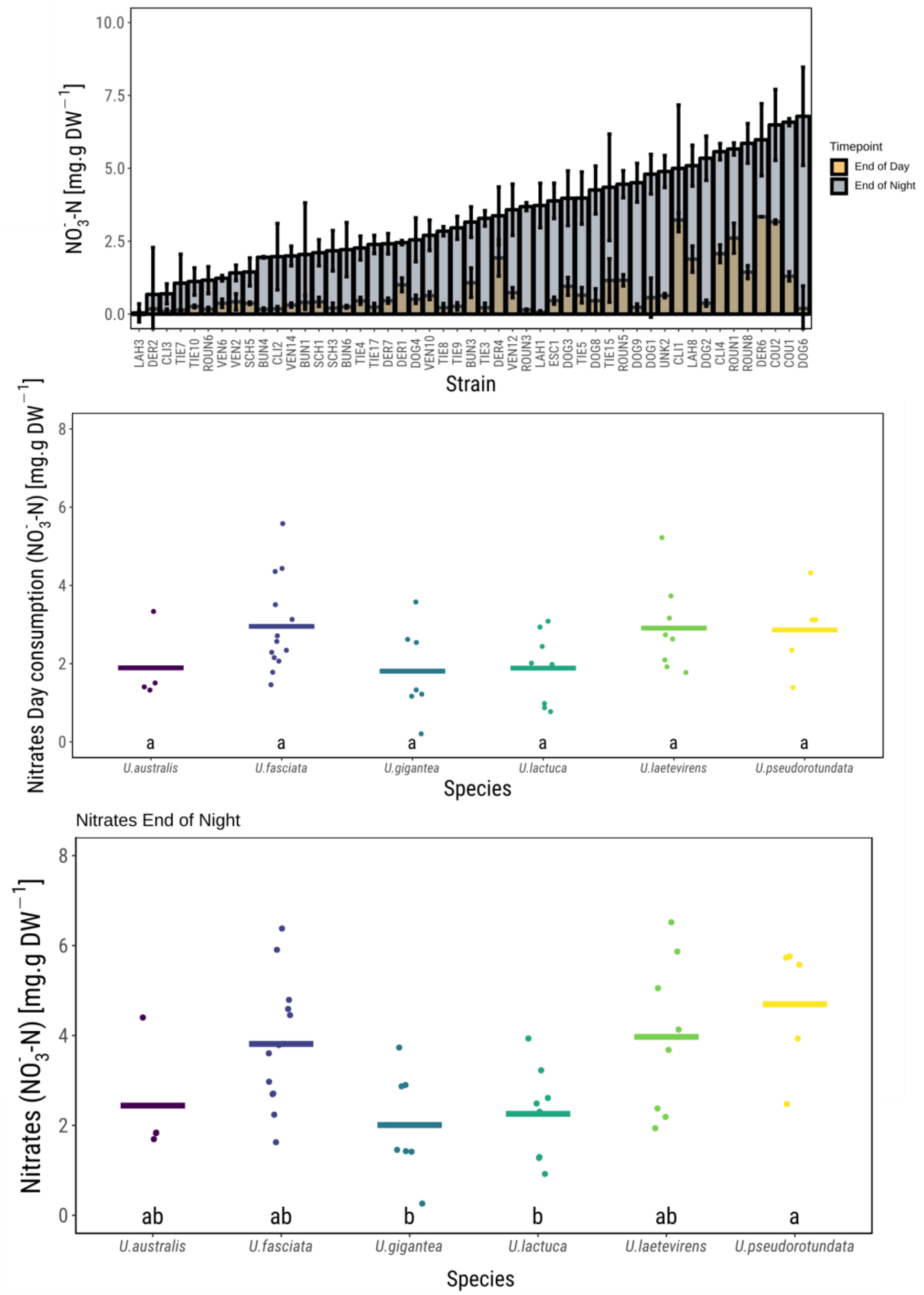

Supplemental Figure S5: Nitrate accumulation in *Ulva* tissue. Top: Nitrate concentration in *Ulva* tissues at the end of day and end of night. Bars represent the mean ± s.d, n = 3 pools of three discs each per timepoint. Middle: Average nitrate consumption during the day for each *Ulva* strains (dots), separated by species. The horizontal line represents the mean per species. Letters represent significance groups (One way ANOVA, $p < 0.05$). Bottom: Average nitrate concentration in tissue for each *Ulva* strains (dots) at the end of the night, separated by species. The horizontal line represents the mean per species. Letters indicate significance groups (Nested ANOVA, $p < 0.05$)

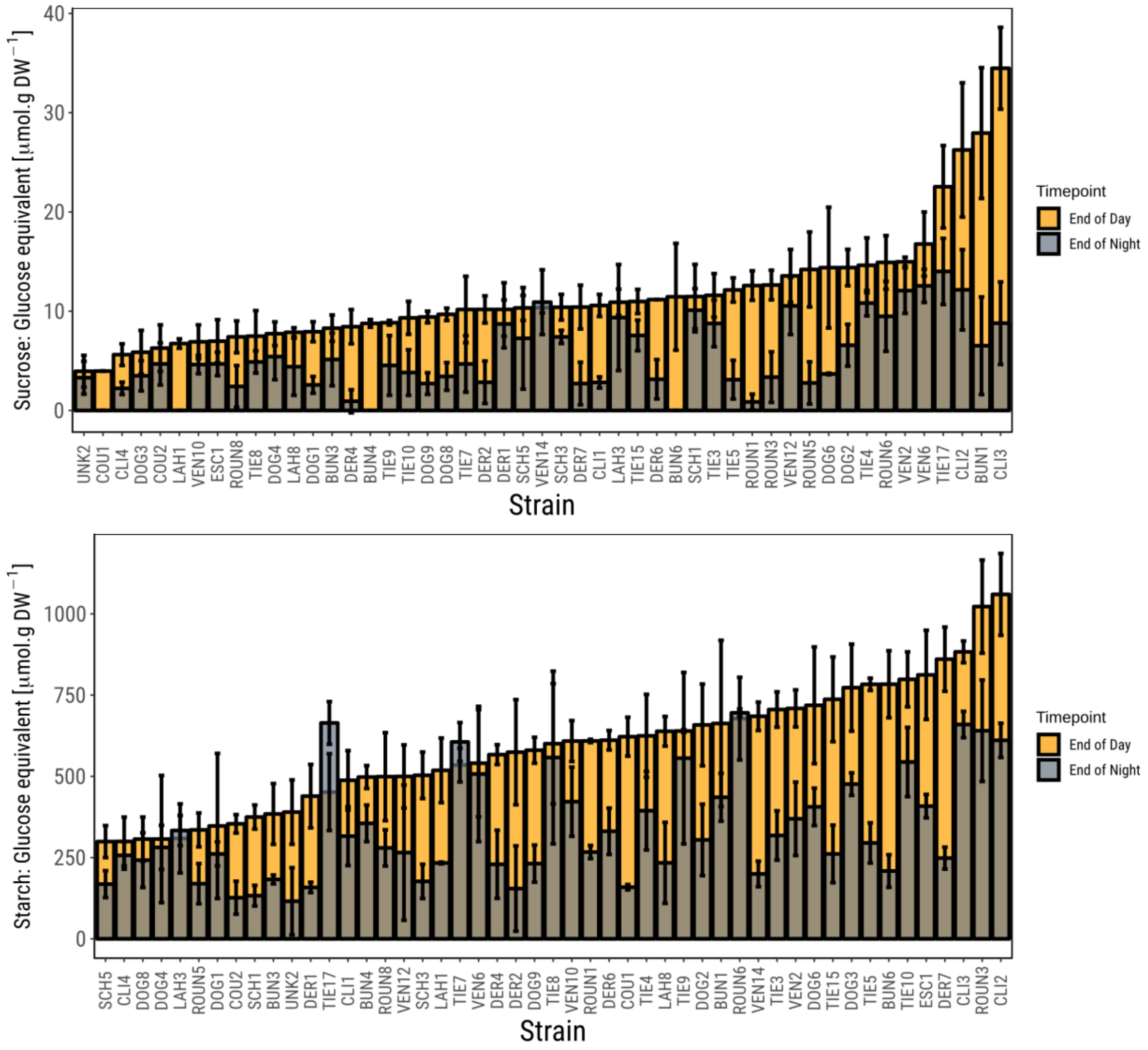

Supplemental Figure S6: Sucrose and starch concentration in *Ulva* tissues at the end of day and end of night. Top: Sucrose concentration in each *Ulva* strain. Bottom: Starch concentration in each *Ulva* strain. Bars represent the mean ± s.d n = 3 pools of three discs each per timepoint.

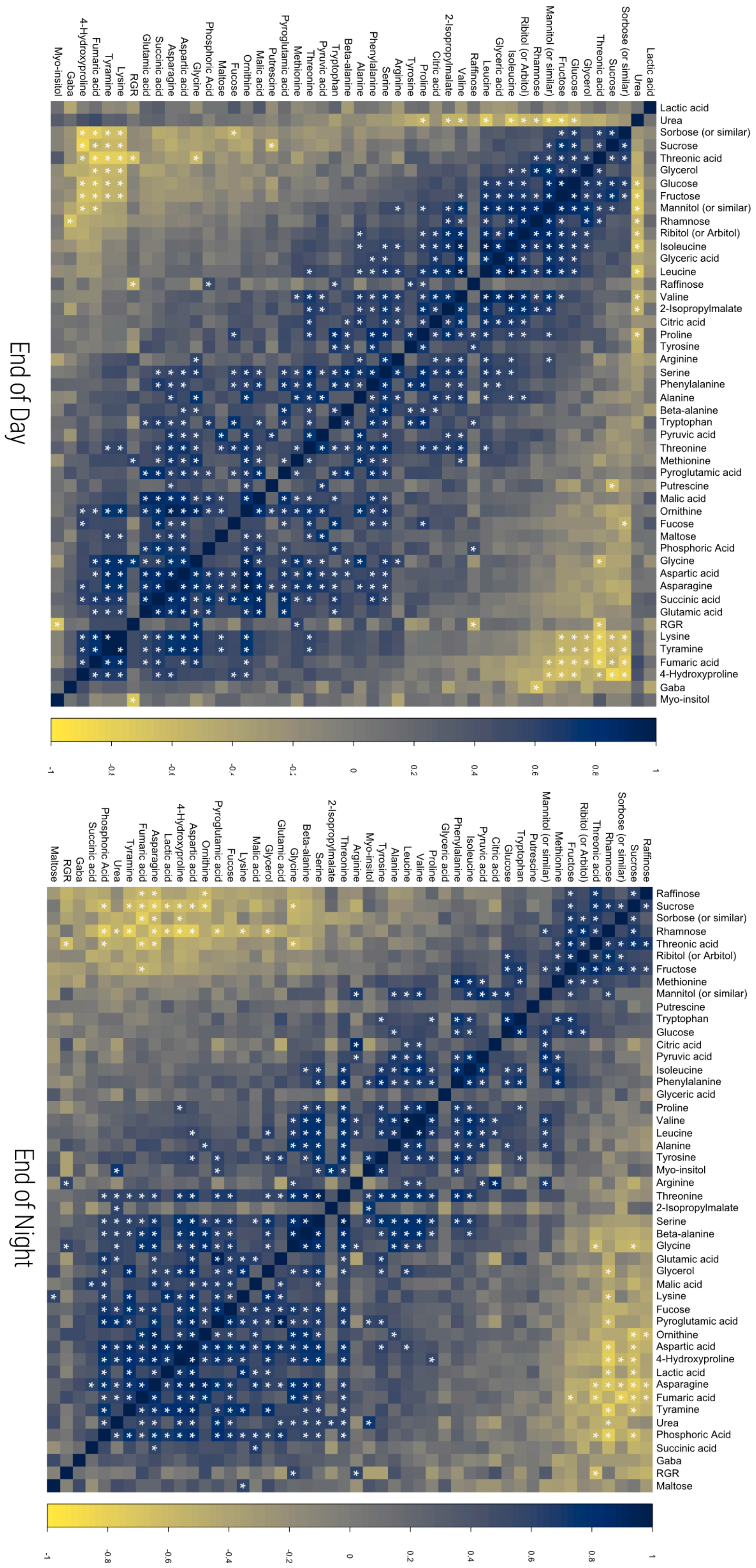
End of Day
End of Night

Supplemental Figure S7: Spearman correlation matrix between metabolites at the end of day and end of night identified by GC-MS. Asterisks represent significant correlations (q-value < 0.05). Data available in Dataset 2.

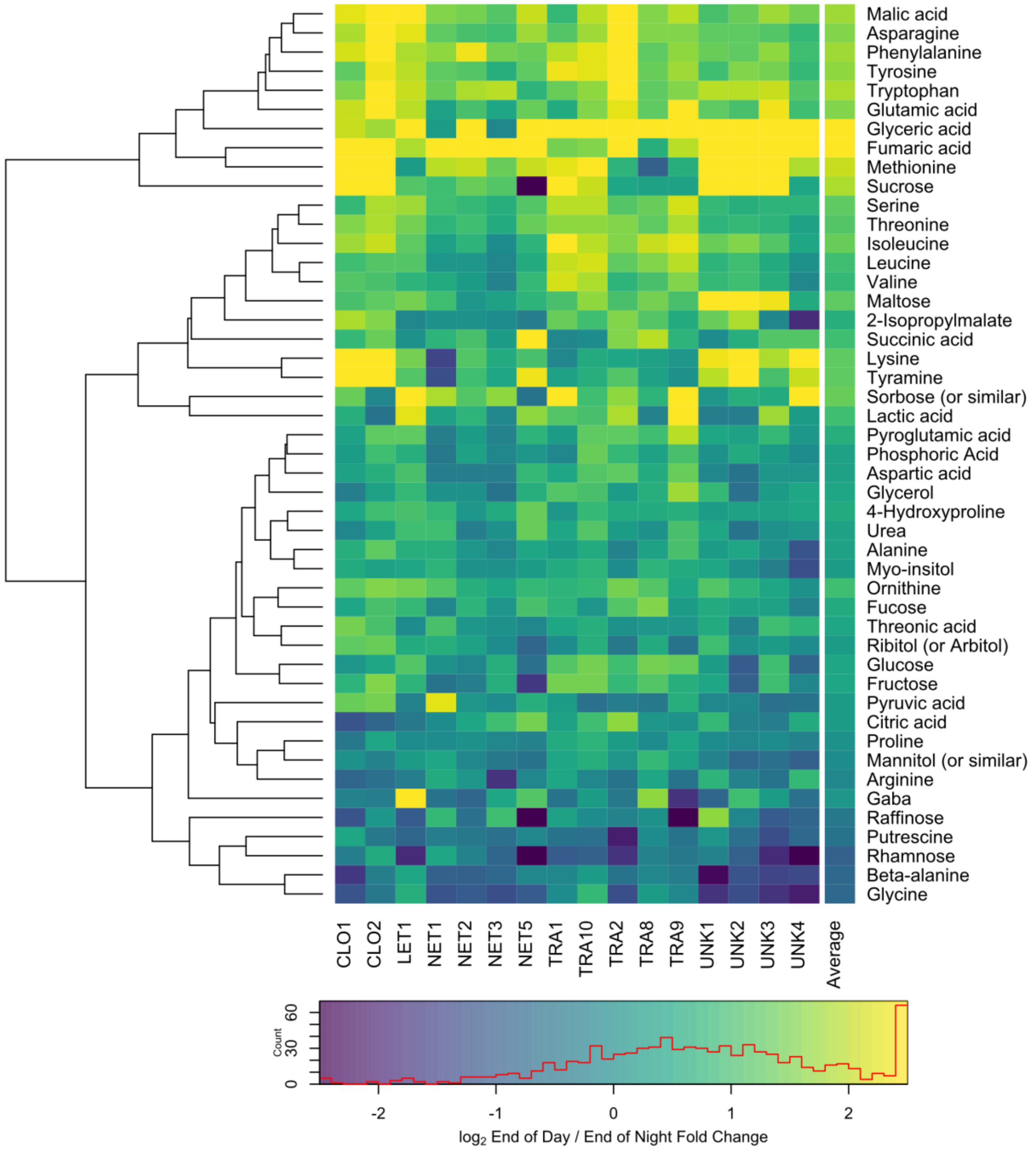

Supplemental Figure S8: Log2 Fold change between the amount of each metabolite at the end of the day and the end of the night. Data available in Dataset 2.

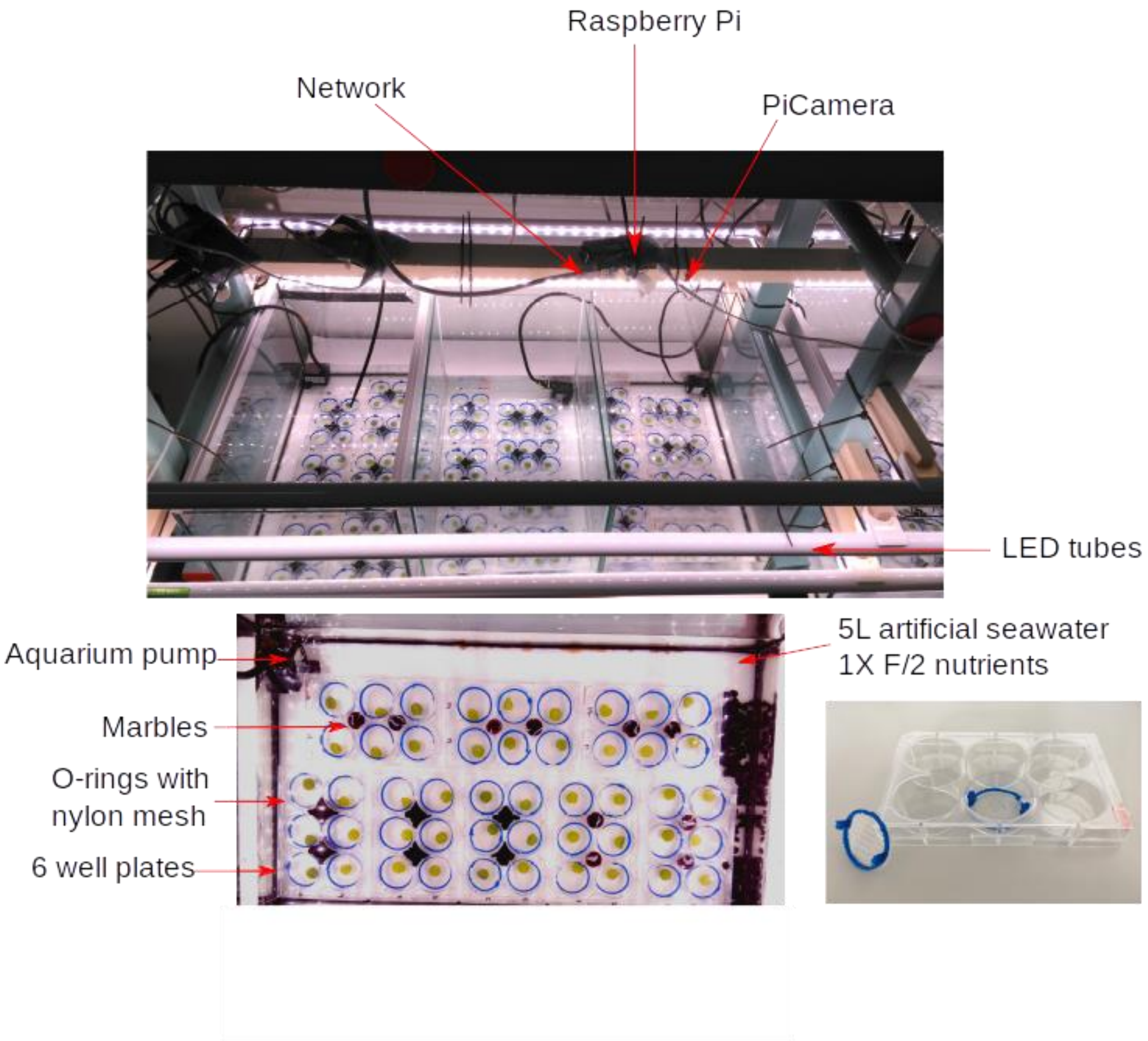

Supplemental Figure S9: Design of the phenotyping platform. Details available in Methods

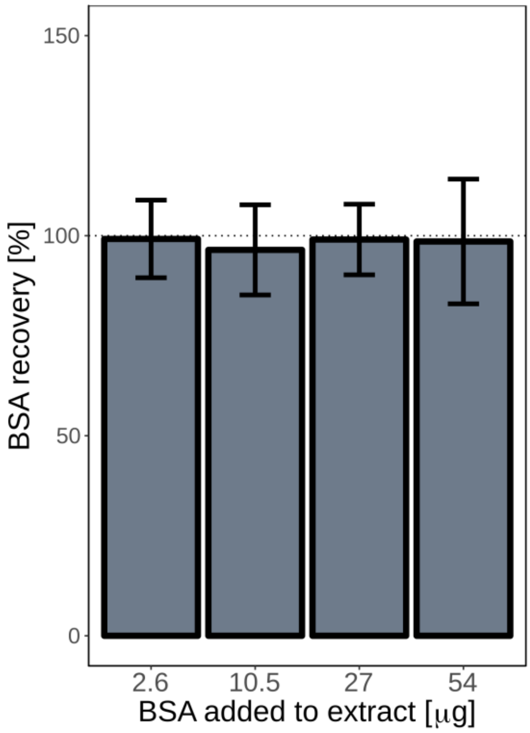

Supplemental Figure S10: The Lowry method is appropriate for protein levels determination in the ethanol-insoluble fraction of *Ulva* tissue. BSA recovery from *Ulva* samples spiked with known amounts of BSA. Data represent the mean ± s.d, n = 8.

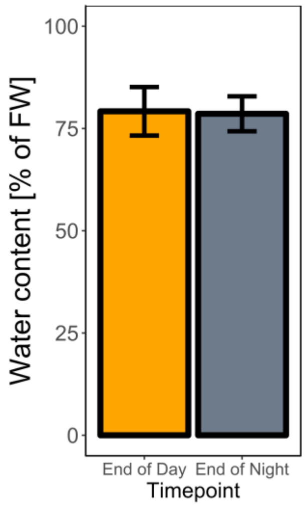

Supplemental Figure S11: Water content of *Ulva* disks at the end of day and end of night. Data represent the mean ± s.d, n = 355 and 367 discs, respectively.
